# 3D-printed PCL/β-TCP/CS composite artificial bone and histocompatibility study

**DOI:** 10.1186/s13018-023-04489-8

**Published:** 2023-12-21

**Authors:** Chao Zheng, Mingman Zhang

**Affiliations:** 1 Department of Orthopaedics, Children’s Hospital of Chongqing Medical University, Chongqing, People’s Republic of China; 2 Department of Pediatric Liver Transplantation, Children’s Hospital of Chongqing Medical University, 136 Zhongshan 2nd Rd., Chongqing, 400014 People’s Republic of China; 3 Ministry of Education Key Laboratory of Child Development and Disorders, National Clinical Research Center for Child Health and Disorders, China International Science and Technology Cooperation Base of Child Development and Critical Disorders, Chongqing Key Laboratory of Pediatrics, Chongqing Engineering Research Center of Stem Cell Therapy, Children’s Hospital of Chongqing Medical University, Chongqing, People’s Republic of China

**Keywords:** Bone defect, Artificial bone, Calcium sulfate, Osteogenesis, 3D printing scaffolds

## Abstract

**Background:**

Tissue-engineered bone materials are an effective tool to repair bone defects. In this study, a novel biodegradable polycaprolactone (PCL)/β-tricalcium phosphate (β-TCP)/calcium sulfate (CS) composite scaffold was prepared by using three-dimensional (3D) printing technology.

**Methods:**

Scanning electron microscopy, gas expansion displacement, and contact goniometry were used to examine the 3D-printed PCL/β-TCP/CS composite scaffolds. The results showed that the PCL/β-TCP/CS scaffolds possessed controllable porosity, hydrophobicity, biodegradability, and suitable apatite mineralization ability. To confirm the bone regenerative properties of the fabricated composite scaffolds, scaffold extracts were prepared and evaluated for their cytotoxicity to bone marrow mesenchymal stem cells (BMSCs) and their ability to induce and osteogenic differentiation in BMSCs.

**Results:**

The PCL/β-TCP/CS composite scaffolds induced a higher level of differentiation of BMSCs than the PCL scaffolds, which occurred through the expression of bone metastasis-related genes. The New Zealand white rabbit radial defect experiment further demonstrated that PCL/β-TCP/CS scaffolds could promote bone regeneration.

**Conclusions:**

In summary, the 3D-printed PCL/β-TCP/CS composite porous artificial bone has good cytocompatibility, osteoinductivity, and histocompatibility, which make it an ideal bone material for tissue engineering.

## Background

Bone defects are common clinical traumas, and large bone defects present a clinical challenge. Traditional treatments include autologous [1] and allogeneic [2] bone grafting; however, they are limited by safety issues such as immune rejection and cross-infection. The rapid development of tissue-engineered bone provides a new way to repair bone defects [3]. Bone tissue engineering research can provide scaffolds for cell transplantation and guide new bone growth [4]. Ideal bone scaffolds should have good biocompatibility and biodegradability, an appropriate degree of mechanical strength, the ability to provide support for new tissues, and have osteoconductivity and osteoinductivity, which promote bone deposition and bone growth [5]. Bone tissue engineering scaffolds are used to induce bone formation and vascularization, and they have a 3D porous structure with a suitable pore size for loading [6]. To improve the mechanical properties of scaffolds and accelerate the rate of osteogenesis, other substances are often introduced to improve their properties, resulting in a variety of bone tissue engineering scaffolds constructed from composite materials.

In recent years, synthetic biomaterials have been used as bone graft substitutes [7]. Polycaprolactone (PCL) is a polymeric organic polymer made by ring-opening polymerization of ε-caprolactone. PCL has good biocompatibility, biodegradability, and good rheology and is widely used in bone tissue engineering. However, its mechanical properties are unremarkable, and its degradation time in vivo is longer than ideal [8, 9]. β-Tricalcium phosphate (β-TCP), which is similar to inorganic components of bone tissue, has good biocompatibility and can induce osteogenesis. The release of calcium and phosphorus ions after degradation can also promote the formation of new bone, but its degradation rate is also slow [10]. Calcium sulfate is a commonly used bone cement with high biocompatibility, advantageous osteoconductivity, and a fast degradation rate [11]. It is difficult for a single material to meet the needs of bone tissue engineering, so research on composite materials is an inevitable trend.

The gradual maturity of 3D printing technology has made it possible to rapidly design and prepare an artificial scaffold with a precise geometry, pore size distribution, and porosity, which can be used to prepare a personalized artificial bone scaffold according to the clinical needs for scaffolds of different sizes, shapes, and volumes, with high accuracy and reproducibility, meeting nearly all the requirements of an ideal scaffold [12]. In this study, we constructed PCL/β-TCP/CS composite scaffolds by 3D printing and verified their histocompatibility and osteogenic differentiation ability.

## Materials and methods

### Preparation of PCL/β-TCP/CS composite scaffold

A 3D printing method based on fused deposition molding technology was used to prepare the scaffold. Computer-aided design (CAD) software was used to design the 3D structure of the scaffold material: a hollow cylindrical structure with an outer diameter of 4.9 mm, an inner diameter of 2.0 mm, a length of 1.5 cm, a pore size of 400 μm, and a porosity of 60%. Then, the 3D model was converted to STL format and imported into a 3D printer (Model: Bio-Architect-Lite, Recongene Biomedical, Nanjing, China). Polycaprolactone (PCL, Mw = 60,000, eSUN New Material, Shenzhen, China), tricalcium β-phosphate (β-TCP, *d*50 = 0.2–5.0 μm 99% pure, EMPEROR Nanomaterials, Nanjing), and calcium sulfate (CS, AR, 97.0%, MACKLIN Biochemical, Shanghai, China) were weighed proportionally and slowly added to chloroform, and the ratios of PCL, β-TCP and CS were 60:20:20, 70:15:15, and 80:10:10:10 (abbreviated as P/T/S20, P/T/S15, and P/T/S10, respectively). The reagents were dissolved in a blender and stirred overnight. The material was dried and cut into granules, added to the extrusion head to test extrusion, and adjusted for stability. The specific parameters of the printer were nozzle diameter 200 μm and speed 2 mm/s. The prepared scaffolding program was run with a stainless-steel bar to fabricate the scaffolding and allow cooling between each layer, and the complete scaffold was allowed to cool and then removed for processing. The PCL/β-TCP/CS composite artificial bone was immersed in ethanol and sterilized by UV, making it ready for use.

### Characterization of the PCL/β-TCP/CS composite scaffold

Microscopy images were captured using a scanning electron microscope (SEM, JSM-7900F, Nippon Electron Corporation JEOL, Japan) to observe the microstructure of the fabricated scaffolds. Four microliters of triple-distilled water was dripped vertically from the feeder to the surface of the scaffold at room temperature, and the static contact angle of the scaffold (LAUDA ScientificOCA20, Germany) was determined using a contact angle meter. The porosity and density of the scaffolds (BSD-PS1/2, BSD Instrument Technology, Beijing, China) were measured using a fully automatic specific surface and pore size analyzer. The measurement principle is the gas expansion displacement method, i.e., using gas molecules with a known molecular cross-sectional area as probes covering the whole surface of the scaffolds to be tested by adsorption and then multiplying the number of adsorbed molecules by the molecular cross-sectional area, which is regarded as the specific surface area of the sample, to derive the actual volume of the scaffold. Porosity = 1 − actual volume of the scaffold/appearance volume of the scaffold; density = mass of the scaffold/actual volume of the scaffold.

### Preparation of PCL/β-TCP/CS composite scaffold infiltration solution

The scaffolds were infiltrated with DMEM (C11885500BT, Gibco, Grand Island, USA) supplemented with 10% (0.1 g/ml) fetal bovine serum (Gibco, 10,099-141C, Grand Island, USA). After incubation at 37 °C in a 5% CO_2_ incubator for 72 h, the culture solution was aspirated and centrifuged and filtered through a 0.22-μm filter to remove bacteria. Then, the extract was stored in a 4 °C refrigerator.

### Measurement of the toxic effects of the PCL/β-TCP/CS composite scaffold on cells by the CCK8 assay

Trypsin digestion of logarithmically grown BMSCs (MUBMX-01001, Cyagen, Guangzhou, China) was performed, and the cells were counted by a cell counting plate and inoculated into 96-well plates at a concentration of 5 × 10^3^ cells/well, 100 μl per well. The cells were incubated in a CO_2_ incubator for 6–8 h. After the cells were attached to the bottom of the culture plate, 100 μl of the extract was added to the experimental group, and an equal volume of 10% fetal bovine serum medium was added to the control group. 10% CCK-8 reagent (10%, CK04, Dojindo, Japan) was added on days 1, 3, 5, and 7, the OD value of the cells was determined, and the relative cell growth rate was calculated after continuing the incubation for 2 h.

### Induction of osteogenic differentiation by PCL/β-TCP/CS composite scaffold extracts

BMSCs were inoculated into 12-well plates at a density of 1 × 10^4^ cells/well, with 3 replicate wells per group. Cells were cultured until 60%–70% confluence, and then, the medium was replaced with PCL support extract. Half of the medium was changed every 3 days. The induction of early osteogenic differentiation of BMSCs was detected by alkaline phosphatase staining on day 7, and the induction of middle and late osteogenic differentiation was detected by alizarin red staining on day 21. Specifically, BMSCs were fixed with 4% formaldehyde solution for 30 min at room temperature on day 7 and then rinsed twice with PBS. They were then stained with 100 µl of alkaline phosphatase solution (C3206, Beyotime, China) for 2 h at 37 °C. On day 21, BMSCs were fixed with 4% paraformaldehyde solution for 30 min at room temperature, then washed twice with PBS, and stained with 100 µl alizarin red S (C0138, Beyotime, China) for 1 h. Photographs were taken under a microscope.

### Real-time quantitative PCR analysis

To further elucidate the stimulatory effect of PCL/β-TCP/CS scaffolds on osteogenic differentiation,

the typical markers runt-related transcription factor 2 (RUNX2), osteopontin (OPN), and BMP2 were evaluated. BMSCs were inoculated in 12-well plates at a density of 1 × 10^4^ cells/well with 3 replicate wells per group. Gene expression of typical markers of BMSCs was assessed after 7 days of culture. Total RNA was extracted using a nucleic acid kit (G3013, Servicebio, China) and converted to cDNA using a reverse transcription kit (G3337, Servicebio, China). SYBR Green master mix (G3320, Servicebio, China) was used for real-time PCR. β-Actin was used as an internal control. The primers used for real-time PCR are listed in Table 1.

**Table 1:** The primer sequences used for the RT-qPCR analysis

Gene	Forward primer	Reverse primer
β-Actin	5-GTGACGTTGACATCCGTAAAGA	5-GTAACAGTCCGCCTAGAAGCAC
BMP2	5-GCTGACCACCTGAACTCCACT	5-CCTCCACAACCATGTCCTGATAA
RUNX2	5-AGCGGACGAGGCAAGAGTTT	5-AGGCGGGACACCTACTCTCATA
OPN	5-TTCTGATGAACAGTATCCTGATGCC	5-ACTTGACTCATGGCTGCCCTTT

### Histocompatibility of the PCL/β-TCP/CS composite scaffold

Thirty healthy New Zealand Large White rabbits (Laboratory Animal Resource Center, Chongqing Medical University, Chongqing, China) weighing 2.5–3.0 kg, male and female, were selected. This experiment was approved by the Animal Research and Ethics Committee of the Children's Hospital of Chongqing Medical University. After intravenous anesthesia with sodium pentobarbital (30 mg/kg) was effective, a 2–3 cm skin incision was made along the long axis of the lateral radius, centered on the middle radius on the left side, and the skin, subcutaneous tissues, fascia, and muscular tissues were bluntly separated to the periosteum in sequence to fully expose the middle radius. A section of radius approximately 1.5 cm in length was excised by using midget electro-saw, and the severed end was rinsed with saline, filled with composite stent, and sutured layer by layer. The incision was sterilized with iodine and fixed with gauze. The rabbits were placed on warm pads and returned to the cage after awakening, and their physiological signs and recovery were observed. In the first postoperative month, rabbit liver and kidney tissues were removed, fixed in 10% formaldehyde, routinely dehydrated and fixed by paraffin embedding, and routinely sectioned before eosin (HE) staining for histological evaluation. Tissues 1 cm around the stent were removed at postoperative months 3, 6, and 12, fixed with formaldehyde, dehydrated and embedded in paraffin, and then stained with Goldner’s trichrome staining. Safranin O-fast green and Masson staining were performed according to routine protocols. Whole sections were scanned using a full paddle scanner (SQS-120P, Shengqiang Technology, Shenzhen, China), and images were acquired using Image Viewer software.

### Statistical analysis

At least 3 replicates were performed in each group for all experiments. Experimental data are expressed using the mean ± standard deviation. Differences between the two samples were evaluated by the t test. One-way analysis of variance was used to assess between-group differences from GraphPad Prism 9.5 software. The findings were considered significantly different when a *p* value < 0.05 was obtained.

## Results

### A broad view of the PCL/β-TCP/CS composite scaffold

**Fig. 1:**
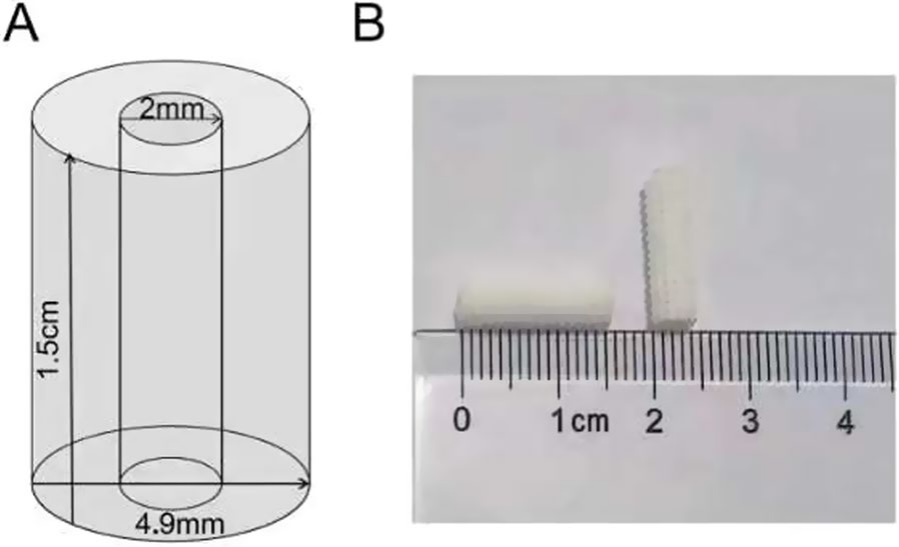
PCL/β-TCP/CS scaffolds. **A** schematic of the scaffold model; **B** a general view of the bone scaffold

Schematic and physical drawings of the PCL/β-TCP/CS scaffolds are shown in Fig. 1. The 3D-printed PCL/β-TCP/CS scaffold is a mesoporous cylindrical structure with an outer diameter of 4.9 mm and a length of 1.5 cm. The artificial bone is white in color, with a rough surface and a uniform texture.

### Physical properties of the PCL/β-TCP/CS composite scaffold

**Fig. 2:**
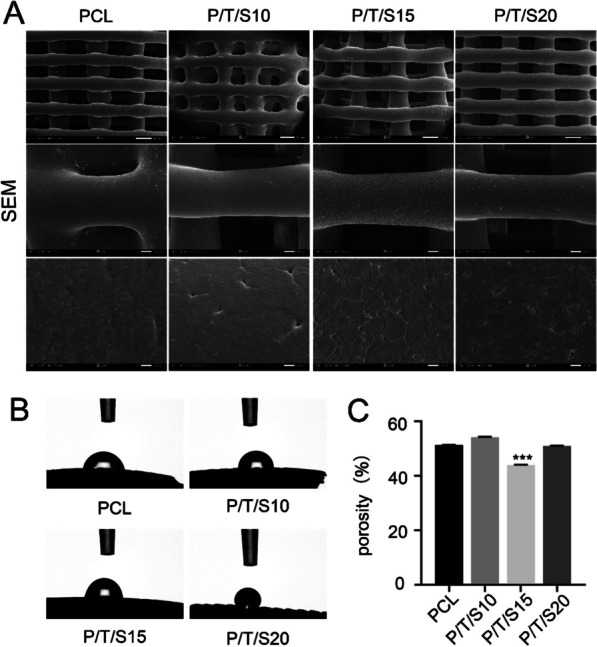
Characterization of PCL/β-TCP/CS scaffold. **A** Scanning electron microscopic images of the PCL/β-TCP/CS composite scaffolds. (scale bar = 500 μm,100 μm,10 μm). **B** The water contact angle of composite scaffolds. **C** The porosity of composite scaffolds of the PCL/β-TCP/CS scaffolds. (***Compared to other groups, *p* < 0.001, *n* = 6)

The microstructure of the PCL/β-TCP/CS composite scaffold is shown in Fig. 2A. Under scanning electron microscopy, the surface of the pure polycaprolactone scaffold was smooth, whereas the addition of β-tricalcium phosphate and calcium sulfate increased the roughness of the scaffold surface, and particles of tricalcium phosphate and calcium sulfate were clearly visible on the surface of the scaffold and in the pores. The pure PCL scaffold and PCL/β-TCP/CS composite scaffolds were filled with interlocking pore structures inside, the pores were interconnected, and the diameter of the micropores was distributed in the range of 400–500 μm.

The porosity of the stent is the key to the exchange of nutrients inside and outside the stent, and a higher porosity ensures that cells inside the stent can obtain enough oxygen and nutrients from the surrounding tissue fluids in the early stage of stent implantation before blood circulation is formed. Figure 2B shows the results of the hydrophilicity experiments for each group of scaffolds. The contact angle of the PCL scaffolds was approximately 78.2°, showing strong hydrophobic surface properties. The contact angle increased slightly after the addition of β-TCP and CS, which was mainly because both β-TCP and CS are hydrophobic materials, and thus, the contact angle increased compared to that of the PCL scaffolds. In addition, the addition of β-TCP and CS roughens the surface of the scaffold, and the roughness increases the hydrophobicity of the surface, so the contact angle increases accordingly. The maximum contact angle was approximately 120.3° for the P/T/S20 group.

Figure 2C shows that the PCL stent had a porosity of 51.28 ± 0.08% due to its plasticity, which was slightly reduced by the addition of β-TCP and CS. The porosity of the three groups of composite stents was as follows: P/T/S20: (50.92 ± 0.11) %; P/T/S15: (43.96 ± 0.11) %; P/T/S10: (54.12 ± 0.15) %. The differences may be mainly due to the decrease in the volume of the overall pores of the scaffold material due to extrusion, which reduces its porosity.

### Cytocompatibility of composite scaffold extracts

**Fig. 3:**
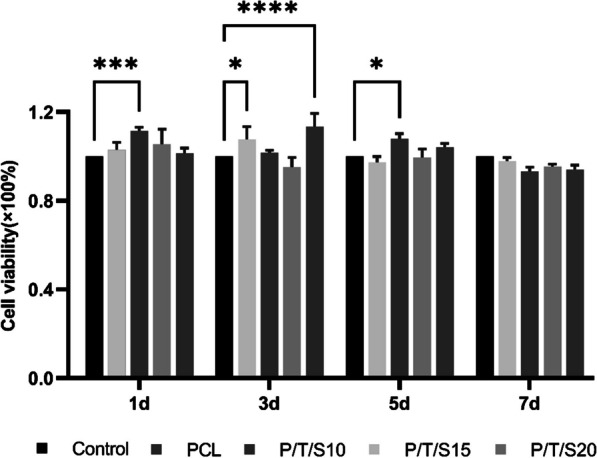
CCK-8 assay for cytotoxicity. (*p < 0.05, *** *p* < 0.001, **** *p* < 0.001; *n* = 3)

Considering that the shape and structure of the scaffolds may affect cells, we tested the biocompatibility of the scaffold materials by determining the toxic effect of the scaffold extract solution on cells. The greater the toxicity of the scaffold extract was, the lower the relative cell survival rate and the lower the absorbance. As shown in Fig. 3, the cell survival rate of each experimental group was above 90%, and the toxicity grade was 0 or 1 according to the national standard GB/T14233.2–2005, which indicated that the composite bone scaffold extract had no toxic components and no toxic inhibition on BMSCs cells, and the scaffold had satisfactory biocompatibility.

### PCL/β-TCP/CS composite scaffold extracts and in vitro osteogenic differentiation potential of mouse BMSCs

**Fig. 4:**
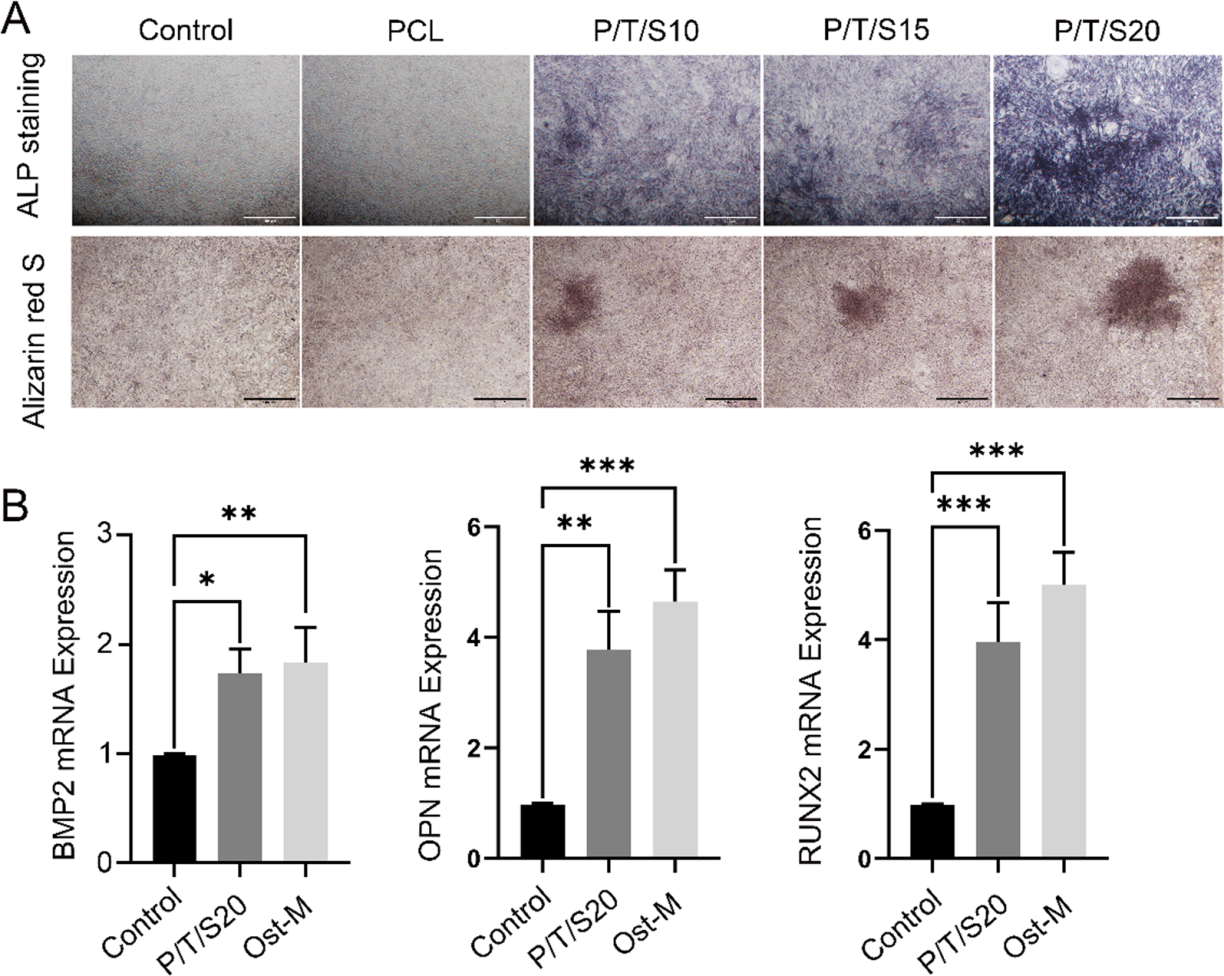
The osteogenic differentiation of BMSCs. **A** BMSCs were cultured in scaffolding solution for 7 and 21 days. Alkaline phosphatase and alizarin red S staining were detected with a scanner. (scale bar = 100 μm). **B** Bone marrow MSCs were cultured in a P/T/S20 scaffold solution containing P/T/S20 for 14 days. Expression of stromal gene mRNA was detected by RT-PCR. mRNA levels were normalized according to the level of the *β*-actin reference gene. Data are means of triplicate analyses. Differences between the control and P/T/S20 groups were significant at the *p < 0.05, **p < 0.01, *** *p < 0.001. (*n* = 3, Ost-M: Osteogenic medium.)

Alkaline phosphatase is an early indicator of cellular osteogenic differentiation, as shown in Fig. 4A. ALP staining was performed in all groups of BMSCs after induction of differentiation to Day 7. Almost no blue-stained cells were observed in the PCL group, and some purple‒blue-stained cells were observed in the PCL/β-TCP/CS composite scaffold group, with a higher number of blue-stained cells in the P/T/S20 group.

Calcium salt deposition is an indicator of the more advanced stages of cellular osteogenic differentiation, and alizarin red formed an orange‒red complex by chelating the calcium ions deposited in the late stage. As shown in Fig. 4A, upon alizarin red staining of the BMSCs on the 21st day of induction of differentiation, almost no orange‒red precipitation was seen in the Control group and PCL group, whereas some orange‒red precipitation was seen in the PCL/β-TCP/CS composite scaffold groups. The staining was more obvious in the P/T/S20 group than in the rest of the groups, which was consistent with the results of alkaline phosphatase staining, suggesting that the P/T/S20 group had the strongest osteoinductive activity.

To further clarify the stimulatory effect of PCL/β-TCP/CS scaffolds on the differentiation of mouse BMSCs, we examined the expression of relevant genes after cell induction by real-time quantitative PCR in scaffold extracts. RT‒PCR analysis showed that the P/T/S20 group promoted the expression of BMP2, RUNX2, and OPN at the mRNA level compared with that in the control group over the course of 7 days of culture (Fig. 4B).

### PCL/β-TCP/CS scaffolds promote bone regeneration in vivo

**Fig. 5:**
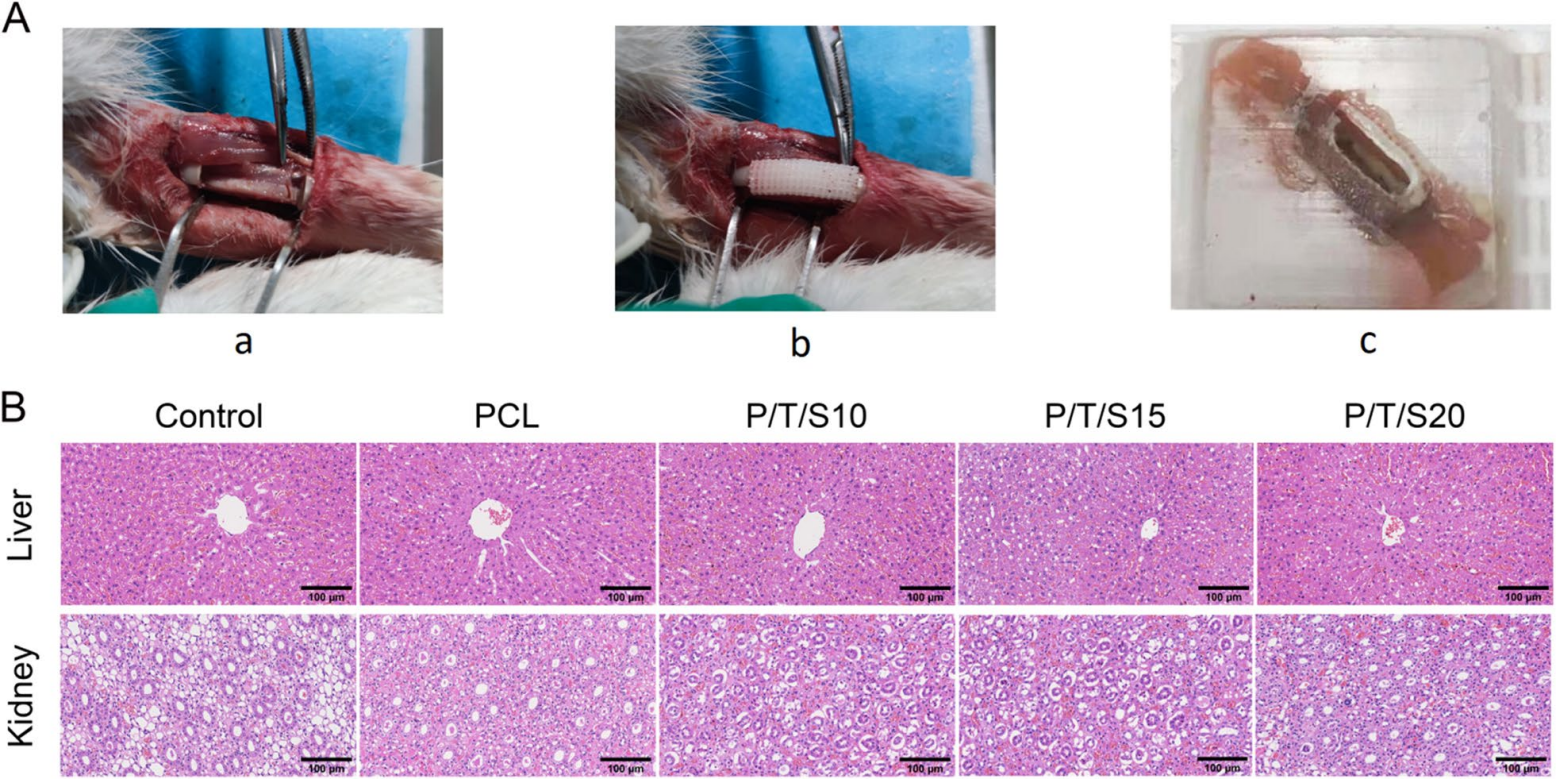
**A** Schematic diagram of operation and sampling. **a** Removal of the radius section; b:Implantation of the composite scaffold; c Paraffin block of scaffold, surrounding muscle and radius tissue. **B** HE staining of liver and kidney tissue Sects. "Background" month after stent implantation in the radial defect. (scale bar: 100um)

Based on the results of the in vitro study, we further investigated the in vivo osteogenic function of the PCL/β-TCP/CS scaffold using the New Zealand rabbit tibial defect model (Fig. 5A). The in vivo toxic effects of the scaffolds were observed by HE staining of the liver and kidney of New Zealand rabbits at 1 month postoperatively (Fig. 5B).

**Fig. 6:**
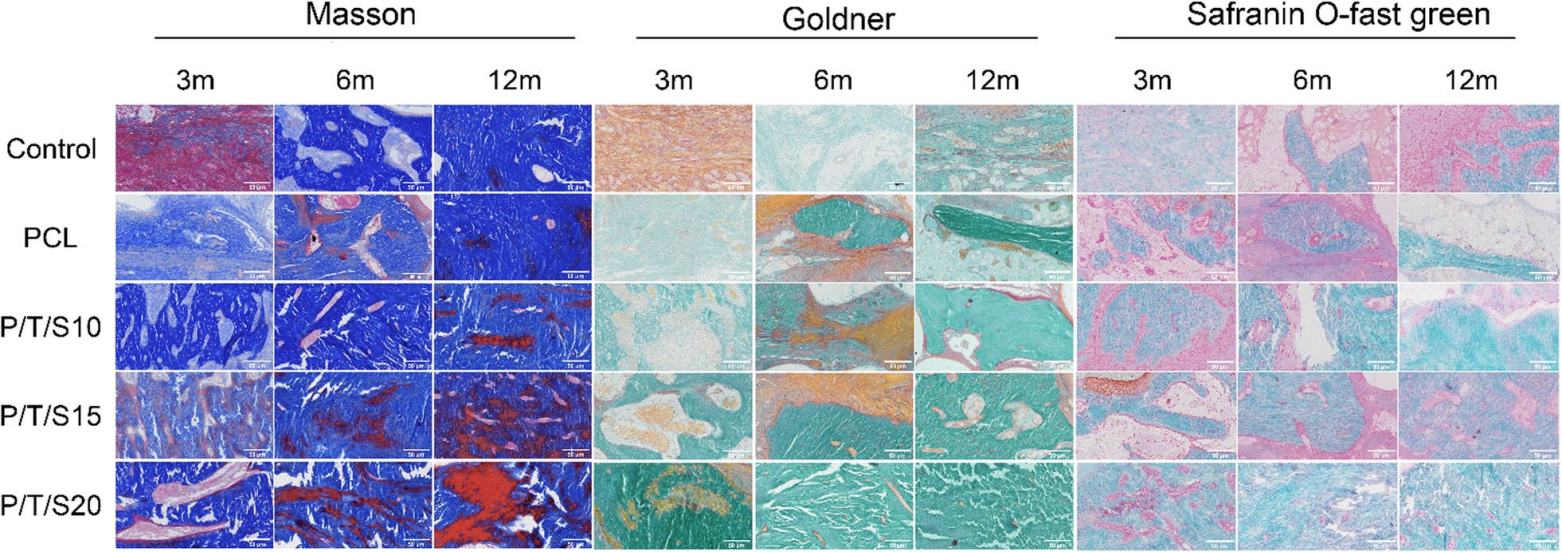
Masson staining, Goldner’s trichrome staining, and Safranin O-fast green staining were observed in each group at each postoperative time point (Scale bar: 50 μm). Masson staining showed new collagen fibers in blue and mature bone in red. Goldner’s trichrome staining showed mineralized bone in green and immature bone in orange–red. Safranin O-fast green staining shows cartilage as red and bone as blue

The results showed that the liver and kidney tissues were structurally normal, with intact hepatic lobules, intact renal units, no hemorrhagic necrosis, and no inflammatory cell infiltration, suggesting that the scaffolds had no effect on the function of the liver and kidneys. Masson staining showed that the new collagen fibers of the bone were blue and that the mature bone was red. Goldner’s trichrome staining showed mineralized bone in green and immature bone in orange‒red. Safranin O-fast green staining showed cartilage in red and bone in blue. The three stains showed an overall trend of increased amounts of newly formed bone in the composite scaffolds in each group, but significant differences between groups remained (Fig. 6). At 3 months postoperatively, bone collagen fibers and bone trabeculae gradually increased in the scaffold group, and bone tissue gradually matured in the P/T/S20 group. At 6 months postoperatively, the immature bone tissue in the control group was higher than that at 4 weeks, the mature bone tissue in the scaffold group increased compared with that at 3 months, and the mature bone tissue in the P/T/S20 group was more abundant. At 12 months postoperatively, there was a small amount of mature bone tissue in the control group; the scaffold composed of mature bone tissue was higher than that at 6 months, and the P/T/S20 group had the most mature bone tissue.

## Discussion

Currently, more than 2 million bone grafts are performed annually in the USA to treat bone defects [13], and bone tissue engineering techniques are currently the primary strategy for treating this problem [14, 15]. From traditional autologous bone grafts and allogeneic bone grafts to the recent rapid development of tissue-engineered artificial bone, an increasing number of bone grafts are being used to promote bone healing and regeneration. Compared to traditional treatments, tissue engineering techniques possess the characteristics of faster fabrication and personalization, and the use of scaffolds in combination with growth factors can further promote the healing of bone defects [16, 17]. 3D printing technology emerged in the 1990s and has proven to be a critical method of manufacturing bone scaffolds [18]. In this study, porous scaffolds with different PCL concentrations were successfully fabricated by 3D bioprinting. The pore size was controlled at 400–500 μm. It has been reported that both pore size and pore volume play important roles in both scaffold degradation and bone healing processes. This is because a large surface area can provide enough living space for cell adhesion and growth, gas and metabolite exchange, angiogenesis, and mineralization [19]. PCL-only scaffolds shave high hydrophobicity and low mechanical properties and must be modified to enhance the scaffold performance. β-TCP and CS are biocompatible inorganic materials with osteoinductive and degradation properties that can improve the roughness of PCL and provide conditions suitable for the adhesion and proliferation of cells. The addition of β-TCP and CS enhanced the toughness of the composite scaffolds and reduced the deformation of PCL, thus improving the mechanical strength of the composite scaffolds [20]. High porosity ranging from 51.7% to 61.6% is considered to be a sign of good interconnection of porous structures, and high porosity is favorable for cell migration and nutrient exchange. The SEM results in this paper showed that the microstructure of PCL/β-TCP/CS scaffolds changed from porous to dense, and at the same time, the porosity and water-absorbing swelling of the scaffolds decreased.

Ideal bone scaffold materials should have good biocompatibility, i.e., should be nontoxic to tissues after implantation. The CCK-8 assay results showed that the survival rate of cells cultured with scaffold extracts in all groups was more than 90%, which demonstrated satisfactory biosafety at the cellular level. Osteoinductivity is the ability of bone scaffolds to induce seed cells to undergo osteogenic differentiation. The degradation products of the scaffold induced osteogenic differentiation of the surrounding osteogenic precursor cells and accelerated the formation of new bone [21]. In addition, the high porosity and interconnected pore structure of bone scaffolds can promote the repair of bone defects. Alkaline phosphatase (ALP) is an early indicator of osteoblast differentiation and osteoclast maturation and plays a key role in the calcification process in vitro [22]. Early alkaline phosphatase (ALP) staining and mid- to late-stage alizarin red staining studies showed that PCL/β-TCP/CS scaffolds induced the osteogenic differentiation of mouse BMSCs without the need for osteoinductive medium, whereas P/T/S20 promoted the osteogenic differentiation of BMSCs even more effectively. RUNX2 regulates chondrocyte and osteoblast differentiation as well as extracellular matrix (ECM) proteins (including BMP2, RUNX2, and OPN) [23]. Real-time PCR results showed that the P/T/S20 scaffold upregulated the expression of osteogenesis-related genes, indicating that the scaffold has the potential to induce the osteogenic differentiation of BMSCs. To explore the potential clinical applications of the PCL/β-TCP/CS scaffold, the researchers created radial defects to compare bone regeneration after the implantation of four PCL/β-TCP/CS scaffolds. In the early stage of stent implantation, the blood circulation has not yet been formed, so local degradation products tend to accumulate, resulting in tissue inflammatory reactions. Since vascular circulation can be formed as early as 4 weeks, we chose to observe the histocompatibility of the stent at 4 weeks after implantation. The results of liver and kidney HE staining also did not show any obvious abnormalities at 4 weeks. Masson and Goldner’s trichrome staining and red solid green staining showed that all PCL/β-TCP/CS scaffold groups significantly promoted bone regeneration compared with the control group, and the P/T/S20 group had the strongest ability to promote bone regeneration.

## Conclusions

In summary, in this study, PCL/β-TCP/CS composite scaffolds were prepared using 3D printing technology. PCL/β-TCP/CS composite scaffolds have high porosity and hydrophobicity, and PCL/β-TCP/CS scaffolds have high apatite mineralization ability, which is conducive to the osteogenic differentiation of BMSCs. When PCL/β-TCP/CS scaffolds were implanted into radial defects of New Zealand rabbits, the P/T/S20 group had significantly better new bone formation and defect healing than the other groups in the 6th and 12th months.

## Data Availability

The datasets analyzed in the study are available from the corresponding author on reasonable request.

## Abbreviations

PCL: Polycaprolactone
β-TCP: β-Tricalcium phosphate
CS: Calcium sulfate
BMSCs: Bone marrow mesenchymal stem cells
CAD: Computer-aided design
RUNX2: Runt-related transcription factor 2
OPN: Osteopontin
ECM: Extracellular matrix
